# Life-long body mass index trajectories and cardiometabolic biomarkers-the Danish diet, cancer, and health-next generations cohort

**DOI:** 10.1038/s41366-025-01882-7

**Published:** 2025-08-22

**Authors:** Jie Zhang, Christina Andersen, Anja Olsen, Jytte Halkjær, Kristina Elin Petersen, Jonas Frey Rosborg Schaarup, Christian S. Antoniussen, Daniel R. Witte, Christina C. Dahm

**Affiliations:** 1 Department of Public Health, Aarhus University, Aarhus, Denmark; 2 Steno Diabetes Center Aarhus, Aarhus, Denmark; 3 Danish Cancer Institute, Copenhagen, Denmark

**Keywords:** Epidemiology, Risk factors

## Abstract

**Background/Objectives:**

Higher body mass index (BMI) is strongly associated with cardiovascular metabolic diseases, however, BMI changes across the lifespan may be complex and non-linear. Furthermore, heterogeneous BMI trajectories may exhibit different cardiometabolic traits. We aimed to identify BMI trajectories over up to 50 years and examine their associations with cardiometabolic biomarkers.

**Subjects/Methods:**

In total, 30,581 participants from the Danish Diet, Cancer and Health - Next Generations cohort were included in the study. Participants recalled their weight history for each decade through questionnaires. Weight and height were measured, and blood samples were collected during a clinic visit. Cardiometabolic biomarkers (Hemoglobin A1c, total cholesterol, triglycerides, high-density lipoprotein, low-density lipoprotein, C-reactive Protein, and creatinine) were determined. Latent class growth models were applied to model BMI trajectories from age 20 until the current age. The optimal number of groups was selected according to Bayesian Information Criteria, the integrated completed likelihood, and the mean posterior probability of each group. Linear and logistic regression models were used to examine the association between distinct BMI trajectories and cardiovascular biomarkers, with adjustment for age, sex, and smoking status.

**Results:**

Four distinct BMI trajectories were identified: “Stable low BMI” group (32%, *n* = 9753), “Gradual BMI increase” (45%, *n* = 13,780), “Early high BMI” group (3%, *n* = 771), and “Steeper BMI increase” group (21%, *n* = 6277). Compared to the “Stable low BMI” group, all other trajectory groups showed significant associations with adverse cardiometabolic biomarkers. For instance, the “Steeper BMI increase” group was associated with elevated triglycerides (β = 0.36 mmol/L, 95% CI: 0.34, 0.38), followed by the “Early high BMI” group (β = 0.30 mmol/L, 95% CI: 0.26, 0.34) and the “Gradual BMI increase” group (β = 0.12 mmol/L, 95% CI: 0.11, 0.13).

**Conclusion:**

Both those with constant high BMI and steeply increased BMI trajectories from age 20 had more unfavorable cardiometabolic profiles compared to those maintaining lower BMI throughout adulthood.

## Introduction

Obesity, commonly measured by body mass index (BMI), is a significant risk factor for cardiovascular diseases (CVD) [1]. Obesity can influence CVD risk directly by inducing alterations in the structure and function of the cardiovascular system, as well as through the effects of adipokines on inflammation and homeostasis [2]. Furthermore, obesity can contribute to the development of CVD by predisposing individuals to subsequent cardiometabolic risk factors, such as insulin resistance, hyperglycemia, hypertension, and dyslipidemia [3].

Previous studies have demonstrated a robust relation between BMI and CVD risk with BMI often measured at a specific point in time during life [4–6]. However, BMI might change substantially across the whole lifespan, and the heterogeneity in the development of BMI may be associated differently with life-long health consequences [7]. In contrast to a single-time measurement, BMI trajectories capture the evolving patterns of weight changes across the life span and might have different implications for health at different ages. Progress has been made in the techniques for modeling the patterns of BMI growth throughout an individual’s entire lifetime, including approaches like latent class growth models (LCGMs) [8], group-based trajectory models [9], and growth mixture models [10]. Among these trajectory methods, LCGMs assume population heterogeneity by identifying distinct subgroups of individuals who follow different developmental trajectories, providing more nuanced insights into these diverse patterns [11]. Moreover, employing models that encompass BMI changes across a person’s entire lifespan can help address the issue of the ‘obesity paradox’ [12]. The paradox might be associated with potential lead time bias [13], wherein individuals with overweight or obesity experience earlier development or earlier diagnosis of cardiovascular diseases compared to those with normal weight.

Recent studies highlight that BMI trajectory groups are differentially associated with cardiometabolic risk factors, including hypertension [14, 15], diabetes, and more adverse lipid profiles [16]. These findings suggest that cardiometabolic risk is dependent not only on the current BMI but also on the individual’s BMI history (i.e., timing of obesity onset and duration). However, the influence of life-long patterns in the accumulation of excessive adiposity on the development of cardiometabolic diseases has yet to be comprehensively understood, as prior investigations have primarily concentrated on BMI trajectories within narrow age groups [17–19]. Weight fluctuations during decades of adulthood could differ from those arising during early life stages, as may their associations with CVD risk.

In the present study, we aimed to identify BMI trajectory patterns across an up to 50-year age period and examine how these BMI trajectory groups are associated with cardiometabolic risk factors using data from a population-based cohort study in Denmark. There are two perspectives of the project: (i) to identify BMI trajectory patterns across adulthood (age 20–70 years); and (ii) to examine the associations between BMI trajectory groups with CVD risk factors. We hypothesized that there was heterogeneity of BMI trajectories across adulthood, and these different BMI trajectories were associated differently with cardiometabolic risk in late adulthood.

## Materials/subjects and methods

### Study population

The study included 30,581 participants from the Danish Diet, Cancer and Health–Next Generations (DCH-NG) cohort. The Diet, Cancer and Health cohort is a population-based cohort, which has been previously described in detail [20]. In 2015–2019, children of Diet, Cancer, and Health cohort members (G1), their spouses (G1P), and grandchildren (G2) were invited to participate in the DCH-NG study, which aimed to investigate associations between genes, diet, and lifestyle across generations [21]. 255, 608 were identified and 197,639 fulfilled the inclusion criteria. 13,875 descendants were excluded due to their status in the CPR (civil registration system, e.g., hidden address or inactive status in CPR). A total of 183,764 individuals were invited by letter and 44,869 agreed to participate in the DCH-NG. Among them, 5315 did not complete questionnaires or have study center assessments, 8933 were excluded due to fewer than 2 recalled BMI values (mean age less than 30 years) or older than 70 years, and 40 were further excluded due to pregnant status or withdrawal of consent. A total of 30,581 participants were eventually included in the analytical sample (Fig. S1).

### BMI and weight history

Participants were invited to complete a physical examination in a study center in Copenhagen or Aarhus. Height, weight, and waist circumference (WC) were measured by trained and certified health personnel. Anthropometric information was obtained for 99% of the participants. Anthropometric measurements were assessed with participants wearing underwear and being barefoot. Height was measured to the nearest 0.1 centimeter (cm) using a wireless stadiometer. Weight was measured to the nearest 0.01 kilograms using a body composition analyzer. BMI was calculated using the standard formula weight (kg) divided by height squared (m^2^). Based on the measured values, BMI was categorized into 4 categories: underweight (<18.5 kg/m^2^), normal weight (18.5–24.9 kg/m^2^), overweight (25–29.9 kg/m^2^), and obesity (≥30 kg/m^2^) according to WHO reference [22].

Anthropometric information was also collected via a questionnaire. Participants were asked to recall their weight history for every 10 years from age 20 years. For example, if a participant was 55 years old, he would recall weight at 20 y, 30 y, 40 y, and 50 y.

### CVD metabolic biomarkers

A blood sample was collected at the study center. Participants were non-fasting, but were asked not to eat a fatty meal, consume alcohol, use chewing gum, brush teeth or similar within two hours prior to their visit to the study center. Whole blood and lithium-heparin blood samples were taken for upfront analysis performed shortly after the visit to the study center. The following biomarkers were assessed: hemoglobin A1c (HbA1c, mmol/mol), total cholesterol (mmol/L), triglycerides (mmol/L), high-density lipoprotein (HDL, mmol/L), low-density lipoprotein (LDL, mmol/L), C-reactive Protein (CRP, mg/L) and creatinine (μmol/L).

Blood pressure was measured using standardized procedures. Systolic blood pressure (SBP) and diastolic blood pressure (DBP) were measured on the left arm three times after 5-min seated rest using the appropriate cuff size. The lowest values of SBP and DBP were used as the final read. In the present study, hypertension was defined as SBP/DBP ≥140/90 mmHg, self-reported doctor diagnosis of hypertension, or use of BP-lowering medication. Dyslipidemia was defined as having triglycerides >2.0 mmol/l or HDL < 1.0 mmol/l based upon the recommendations by the National Heart Foundation [23] and the Australian Diabetes Society [24]. Glucose metabolism status was determined by the American Diabetes Association criteria based on HbA1c. Prediabetes status was based in the range of 39–46 mmol/mol and cases of type 2 diabetes were defined as ≥48 mmol/mol [25].

### Lifestyle questionnaire

Self-reported smoking information was obtained from a web-based lifestyle questionnaire which was provided to the participants shortly after recruitment. Participants were asked ‘Have you ever smoked?’ and ‘Do you currently smoke?’ We derived the smoking variable and categorized it into three groups: current, former, and never smoker.

### Latent growth models

Participants with 3 or more BMI assessments (usually the measured assessment from study entry and two self-reported weight history measures) were included in the BMI trajectory analyses. LCGM were applied to model the BMI trajectories from age 20 until the current age. The LCGM identifies latent classes of BMI that differ in the initial state and in the way they change over time. Of note, younger participants were less likely to have 3 or more BMI assessments.

All trajectories were modeled under the assumption of equivalent polynomial orders, with model specifications encompassing linear, quadratic, and cubic polynomials. The optimal number of latent classes was determined through a stepwise forward selection procedure, initiating with a single-class solution and sequentially increasing the number of classes up to a maximum of four groups. The optimal number of groups and model pattern were chosen according to changes in Bayesian Information Criteria (BIC), the Integrated Completed Likelihood (ICL), entropy, and the mean posterior probability of each group. We set the criteria that each trajectory group needed to include at least 1% of the participants. The final trajectory shapes were determined using BIC and Wald tests for each age term. After selecting the appropriate model, participants were assigned to latent classes based on the highest posterior class membership probabilities, obtained from the estimated parameters of the LCGM model and their observed responses [26]. In the final model, a cubic function was used, with age, age^2^, and age^3^ terms. We also performed the LCGM models separately for men and women, but the patterns were similar, thus, we used the whole sample in the final analyses.

### BMI growth trajectories and CVD risk

We present population characteristics by BMI growth trajectory groups. Continuous measures are presented as mean ± standard deviation (SD), and median/interquartile range (IQR) for non-normal distributed variables. Categorical variables are shown as percentages. Multivariable linear and logistic regression models were applied to investigate the relationship between BMI growth trajectory membership and CVD risk markers. We started by including BMI growth trajectories groups in the crude model (Model 1); then we adjusted Model 1 for potential confounding factors, including current age, sex, and smoking status (Model 2). We further adjusted for lipid-lowering and antihypertensive drugs in Model 3.

### Stratification analyses

We conducted the analyses stratified by sex to investigate potential effect medication by sex. Linear and logistic regression models were fitted for men and women, separately. We also examined potential effect modification by medication use through stratified analyses. We created separate models for participants based on their medication status: (1) those taking versus not taking lipid-lowering medications; (2) those taking versus not taking antihypertensive medications; and (3) those taking versus not taking either lipid-lowering or antihypertensive medications.

### Sensitivity analyses

We restricted the analyses to participants older than 50 years who provided more complete and homogenous BMI histories, with ideally 4 BMI values to ensure reliable capture of long-term BMI trajectories.

The statistical software packages Stata/IC 16.0 [27] (StataCorp) and R (v4.0.2) [28] were used to conduct the analyses.

## Results

### Latent class trajectories

**Fig. 1:**
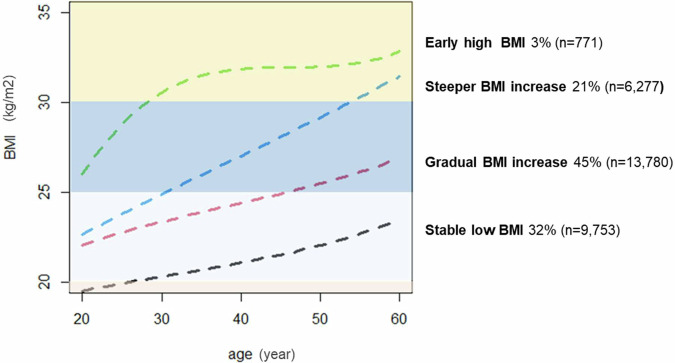
The distinct trajectory patterns of body mass index from early to late adulthood in the DCH-NG Cohort. Four distinct patterns were identified through latent class growth modeling, with the percentage and number of participants in each group indicated. Dashed lines represent class-specific mean predicted BMI trajectories from the best-fitting model; shaded horizontal bands indicate conventional BMI ranges (for reference only). The number and percentage of participants in each latent class are provided in the figure legend. BMI body mass index, DCH-NG diet cancer and health–next generations cohort.

We identified four BMI trajectories from 30,581 participants within the 20–70-year age span. Of these, 32% followed a mean trajectory with slight BMI increase from early adulthood to late adulthood, maintaining BMI values predominantly below 25 kg/m² (‘Stable low BMI’ group, *n* = 9753); 45% were classified into a group characterized by a moderate BMI increase pattern, with mean class trajectories rising from approximately 22 kg/m² in early adulthood to 27 kg/m² by late adulthood (‘Gradual BMI increase’ group, *n* = 13,780); 21% belonged to a class with a more pronounced BMI increase pattern, with mean trajectories starting around 23 kg/m² in early adulthood and rising to approximately 31 kg/m² by late adulthood (‘Steeper BMI increase’ group, *n* = 6277); and 3% were assigned to a class characterized by elevated BMI values from early adulthood (approximately 26 kg/m²), with mean trajectories increasing rapidly above 30 kg/m² by their early 30 s and remaining stable afterwards (‘Early high BMI’ group, *n* = 771). Figure 1 presents the four BMI trajectory groups.

Demographic characteristics and cardiometabolic risk factors according to the four trajectories are presented in Table 1. The average age of participants was 50 years (SD = 7.7 years), while the ‘Early high BMI’ group was relatively young (mean age = 46 years, SD = 7.5years). Participants in the ‘Stable low BMI’, ‘Steeper BMI increase’, and ‘Early high BMI’ trajectory groups were more likely to be female, while those in the ‘Gradual BMI increase’ group were more likely to be male (Table 1). Differences in smoking, BMI, SBP, DBP, triglycerides, total cholesterol, HDL, LDL, and CRP across the four trajectory groups were observed.

**Table 1:** Characteristics of participants by BMI trajectories in the DCH-NG cohort.

Variables, categorical, n(%)		Stable low BMI	Gradual BMI increase	Steeper BMI increase	Early high BMI	Total
		*N* = 9753 (32%)	*N* = 13,780 (45%)	*N* = 6277 (21%)	*N* = 771 (3%)	*N* = 30,581
Sex	Male	2142 (22%)	8163 (59%)	2769 (44%)	228 (30%)	13,302 (43%)
	Female	7611 (78%)	5617 (41%)	3508 (56%)	543 (70%)	17,279 (57%)
Smoking	Current	1494 (15%)	2163 (16%)	1125 (18%)	171 (22%)	4953 (16%)
	Former	3309 (34%)	4530 (33%)	2156 (34%)	255 (33%)	10,250 (34%)
	Never	4950 (51%)	7087 (51%)	2996 (48%)	345 (45%)	15,378 (50%)
BMI Categories based on measured values	Normal	8913 (91%)	5574 (40%)	659 (11%)	100 (13%)	15,246 (50%)
	Overweight	821 (8%)	7611 (55%)	2434 (39%)	222 (29%)	11,088 (36%)
	Obesity	15 (0%)	585 (4%)	3174 (51%)	435 (57%)	4209 (14%)
Generation	G1	6740 (69%)	9374 (68%)	4273 (68%)	509 (66%)	20,896 (68%)
	G1A	2517 (26%)	3531 (26%)	1407 (22%)	139 (18%)	7594 (25%)
	G2	496 (5%)	875 (6%)	597 (10%)	123 (16%)	2091 (7%)
Use of blood pressure lowering medication	Yes	636 (7%)	1316 (10%)	996 (16%)	127 (16%)	3075 (10%)
Use of lipid lowering medication	Yes	335 (3%)	773 (6%)	531 (8%)	59 (6%)	1698 (6%)
***Continuous, Mean (SD)***						
Age (years)		50.7 (7.5)	50.0 (7.8)	48.9 (7.7)	46.0 (7.5)	49.9 (7.7)
BMI (kg/m2)		22.09 (2.05)	25.74 (2.30)	30.21 (4.42)	32.05 (6.50)	25.65 (4.25)
Triglycerides (mmol/L)*		0.03 (0.46)	0.25 (0.53)	0.43 (0.55)	0.31 (0.52)	0.22 (0.53)
Total cholesterol (mmol/L)		5.16 (0.94)	5.23 (0.97)	5.25 (1.01)	5.00 (0.97)	5.21 (0.97)
HDL (mmol/L)		1.80 (0.45)	1.54 (0.18)	1.40 (0.41)	1.44 (0.43)	1.59 (0.46)
LDL (mmol/L)		3.09 (0.84)	3.27 (0.88)	3.31 (0.92)	3.08 (0.90)	3.21 (0.88)
HbA1c (mmol/mol) *		1.64 (0.07)	1.65 (0.07)	1.67 (0.10)	1.67 (0.11)	1.65 (0.08)
CRP* (mg/L)		−0.35 (0.92)	−0.13 (0.93)	0.39 (0.99)	0.50 (1.12)	−0.07 (0.99)
Creatinine (μmol/L)*		4.24 (0.17)	4.33 (0.18)	4.29 (0.20)	4.23 (0.20)	4.29 (0.19)
SBP (mmHg)		114.88 (16.72)	121.35 (16.46)	122.97 (16.66)	120.89 (17.38)	119.61 (16.93)
DBP (mmHg)		80.09 (10.98)	83.69 (10.90)	85.94 (11.04)	85.23 (11.74)	83.04 (11.19)

*BMI* body mass index, *CRP* C-reactive Protein, *DBP* diastolic blood pressure, *HbA1c* hemoglobin A1c, *HDL* high-density lipoprotein HDL, *LDL* low-density lipoprotein, *SBP* systolic blood pressure, *SD* standard deviation, *IQR* interquartile range *log-transformed.

### Association between BMI trajectories and cardiometabolic risk factors

**Fig. 2:**
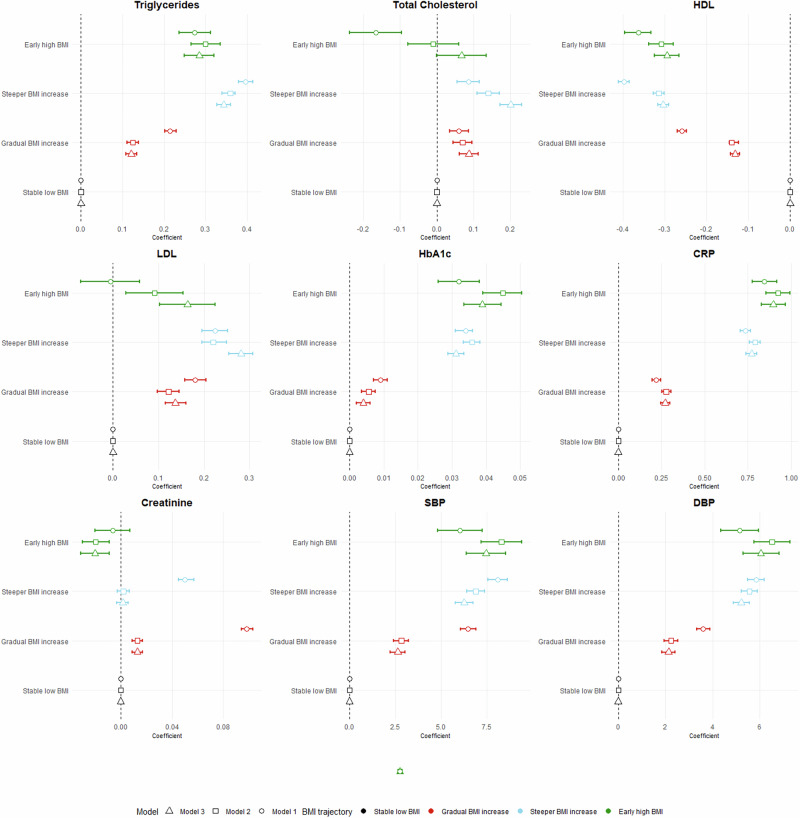
Associations between BMI trajectory classes and cardiometabolic biomarkers in the DCH-NG Cohort. Linear regression models were used to assess associations, with the “Stable normal BMI” trajectory group as the reference category. Model 1: Crude model. Model 2: Adjusted for age, sex, and smoking status. Model 3: Adjusted for age, sex, smoking status, and use of lipid-lowering and antihypertensive medications. Biomarkers assessed: hemoglobin A1c (HbA1c, mmol/mol), total cholesterol (mmol/L), triglycerides (mmol/L), high-density lipoprotein (HDL, mmol/L), low-density lipoprotein (LDL, mmol/L), C-reactive Protein (CRP, mg/L), creatinine (μmol/L), systolic blood pressure (SBP, mmHg), and diastolic blood pressure (DBP, mmHg). Triglycerides, HbA1c, CRP, and creatinine were log-transformed for analysis due to non-normal distribution. BMI body mass index, LCGM latent class growth models, OW overweight.

The four distinct BMI trajectories were associated with cardiometabolic biomarkers in the adjusted linear regression models. The ‘Steeper BMI increase’ group showed stronger associations with unfavorable lipid profiles compared to the ‘Stable low BMI’ group, with marked elevations in triglycerides (β = 0.36 mmol/L, 95% CI: 0.34, 0.38) and reduced HDL cholesterol (β = −0.32 mmol/L, 95% CI: −0.33, −0.30). Similarly, the ‘Early high BMI’ group was associated with elevated triglycerides (β = 0.30 mmol/L, 95% CI: 0.26, 0.34) and decreased HDL (β = −0.31 mmol/L, 95% CI: −0.34, −0.28). The ‘Gradual BMI increase’ group demonstrated modest but still significant associations with triglycerides (β = 0.12 mmol/L, 95% CI: 0.11, 0.13) and HDL (β = −0.14 mmol/L, 95% CI: −0.15, −0.12) (Model 2). For blood pressure measures, the ‘Early high BMI’ group showed the strongest associations, with notably elevated systolic blood pressure (SBP: β = 8.28 mmHg, 95% CI: 7.18, 9.37) and diastolic blood pressure (DBP: β = 6.51 mmHg, 95% CI: 5.74, 7.28) compared to the ‘Stable low BMI’ group. The ‘Steeper BMI increase’ group also demonstrated substantial elevations (SBP: β = 6.88 mmHg, 95% CI: 6.40, 7.36; DBP: β = 5.56 mmHg, 95% CI: 5.22, 5.89), while the ‘Gradual BMI increase’ group showed more modest but still significant associations (SBP: β = 2.82 mmHg, 95% CI: 2.41, 3.23; DBP: β = 2.23 mmHg, 95% CI: 1.95, 2.52). For HbA1c, the magnitude was similar for the ‘Steeper BMI increase’ and ‘Early high BMI’ groups (Fig. 2). Both ‘Graduate BMI increase, ‘Steeper BMI increase’ and ‘Early high BMI’ were associated with elevated CRP levels compared to the ‘Stable low BMI group’. The magnitude of associations increased slightly after further adjusting for lipid-lowering and antihypertensive medications, particularly for lipid profiles, whereas the associations with SBP and DBP were modestly attenuated. Additional data are shown in Table 2.

**Table 2:** Association between BMI trajectory groups and cardiometabolic biomarkers in multivariable regression models.

Models	Trajectory groups	Triglycerides* (mmol/L)		Total Cholesterol (mmol/L)		HDL (mmol/L)		LDL (mmol/L)		HbA1c* (mmol/mol)		CRP* (mg/L)		Creatinine* (μmol/L)		SBP (mmHg)		DBP (mmHg)	
		β	95%CI	β	95%CI	β	95%CI	β	95%CI	β	95%CI	β	95%CI	β	95%CI	β	95%CI	β	95%CI
1	Stable low BMI	ref		ref		ref		ref		ref		ref		ref		ref		ref	
	Gradual BMI increase	0.22	0.19,0.25	0.06	0.03,0.09	−0.26	−0.27,−0.25	0.18	0.16,0.20	0.01	0.01,0.01	0.22	0.19,0.25	0.10	0.10,0.10	6.47	6.04,6.90	3.60	3.32,3.88
	Steeper BMI increase	0.40	0.37,0.43	0.09	0.06,0.12	−0.40	−0.41,−0.39	0.22	0.19,0.25	0.03	0.03,0.03	0.74	0.71,077	0.05	0.04,0.06	8.09	7.56,8.62	5.84	5.49,6.19
	Early high BMI	0.27	0.20,0.34	−0.17	−0.24,−0.10	−0.36	−0.39,−0.33	−0.01	−0.07,0.05	0.03	0.02,0.04	0.85	0.78,0.92	−0.01	−0.02,−0.00	6.00	4.78,7.22	5.14	4.33,5.95
2	Stable low BMI	ref		ref		ref		ref		ref		ref		ref		ref		ref	
	Gradual BMI increase	0.12	0.11,0.13	0.07	0.04,0.10	−0.14	−0.15,−0.12	0.12	0.10,0.14	0.01	0.00,0.01	0.28	0.25,0.30	0.01	0.01,0.02	2.82	2.41,3.23	2.23	1.95,2.52
	Steeper BMI increase	0.36	0.34,0.38	0.14	0.11,0.17	−0.32	−0.33,−0.30	0.22	0.19,0.25	0.04	0.03,0.04	0.79	0.76,0.82	0.00	−0.00,0.01	6.88	6.40,7.36	5.56	5.22,5.89
	Early high BMI	0.30	0.26,0.34	−0.01	−0.08,0.06	−0.31	−0.34,−0.28	0.09	0.03,0.15	0.04	0.04,0.05	0.92	0.85,0.99	−0.02	−0.03,−0.01	8.28	7.18,9.37	6.51	5.74,7.28
3	Stable low BMI	ref		ref		ref		ref		ref		ref		ref		ref		ref	
	Gradual BMI increase	0.12	0.11,0.13	0.09	0.06,0.11	−0.13	−0.14,−0.12	0.14	0.11,0.16	0.00	0.00,0.00	0.27	0.24,0.30	0.01	0.01,0.02	2.63	2.23,3.04	2.13	1.84,2.41
	Steeper BMI increase	0.34	0.33,0.36	0.20	0.17,0.23	−0.30	−0.32,−0.29	0.28	0.25,0.31	0.03	0.03,0.03	0.77	0.74,0.80	0.00	−0.00,0.01	6.25	5.76,6.73	5.21	4.87,5.55
	Early high BMI	0.29	0.25,0.32	0.07	0.00,0.13	−0.30	−0.33,−0.27	0.16	0.10,0.23	0.04	0.03,0.04	0.90	0.83,0.97	−0.02	−0.03,−0.01	7.44	6.35,8.53	6.04	5.28,6.81

Multivariable linear models between BMI trajectory groups and cardiometabolic biomarkers, with stable low BMI as reference group.

Mode1 crude model; Model 2 was adjusted for age, sex, and smoking; Model 3 was adjusted for age, sex, smoking, and medication.

*log-transformed.

*BMI* body mass index, *CI* confidence interval, *CRP* C-reactive protein, *DBP* diastolic blood pressure, *HbA1c* hemoglobin A1c, *HDL* high-density HDL, *LDL* low-density lipoprotein, *SBP* systolic blood pressure.

In Table 3, we present the ORs between BMI trajectories and hypertension, dyslipidemia, and pre-diabetes. Compared with the ‘Stable low BMI’ group, the ORs of dyslipidemia for the ‘Gradual BMI increase’, ‘Steeper BMI increase’, and ‘Early high BMI’ were 2.30 (95% CI: 1.97–2.68), 6.21 (95% CI: 5.33–7.22), and 5.86 (95% CI: 4.53–7.60), respectively. Similarly, compared with the ‘Stable low BMI’ group, the ‘gradual BMI increase’, the ‘Steeper BMI increase’ and ‘Early high BMI’ trajectories group was associated with higher odds of hypertension, with the ORs of 1.43 (95% CI: 1.34–1.53), 2.56 (95% CI: 2.37–2.76) and 3.02 (95% CI: 2.57–3.56), respectively. We also observed the ‘Steeper BMI increase’ and ‘Early high BMI’ groups had 8-fold (95% CI: 6.4–10.3) and 15-fold (95% CI: 10.4–21.5) higher odds of pre-diabetes compared to the ‘Stable low BMI’ (Model 2, Table 3). The ORs showed similar patterns after further adjustment for lipid-lowering and antihypertensive medications, with slight attenuations for hypertension and pre-diabetes (Model 3, Table 3).

**Table 3:** Association between BMI trajectory groups and cardiometabolic risk in logistic regression models.

	Model 1			Model 2			Model 3		
Hypertension	OR	95% CI		OR	95% CI		OR	95% CI	
Stable low BMI	Ref.			Ref.			Ref.		
Gradual BMI increase	1.72	1.62	1.83	1.43	1.34	1.53	1.40	1.31	1.50
Steeper BMI increase	2.44	2.27	2.62	2.56	2.37	2.76	2.39	2.22	2.58
Early high BMI	2.13	1.82	2.49	3.02	2.57	3.56	2.76	2.34	3.26
**Dyslipidemia**									
Stable low BMI	Ref.			Ref.			Ref.		
Gradual BMI increase	2.56	2.20	2.96	2.30	1.97	2.68	2.31	1.99	2.70
Steeper BMI increase	6.39	5.51	7.41	6.21	5.33	7.22	6.37	5.47	7.42
Early high BMI	5.51	4.27	7.11	5.86	4.53	7.60	6.04	4.65	7.83
**Pre-diabetes and diabetes**									
Stable low BMI	Ref.			Ref.			Ref.		
Gradual BMI increase	2.10	1.64	2.69	1.85	1.44	2.39	1.67	1.29	2.16
Steeper BMI increase	7.01	5.53	8.88	8.11	6.36	10.34	6.12	4.76	7.85
Early high BMI	8.34	5.88	11.85	14.97	10.41	21.53	10.37	7.10	15.15

Model 1 was crude model; Model 2 was adjusted for age, sex, and smoking; Model 3 was adjusted for age, sex, smoking, and medication.

Hypertension was defined as systolic/diastolic BP ≥ 140/90 mmHg, self-reported doctor diagnosis of hypertension, or use of BP-lowering medication. Dyslipidemia was defined as having triglycerides >2.0 mmol/l or HDL < 1.0 mmol/l based upon the recommendations by the National Heart Foundation [2] and the Australian Diabetes Society [3]. Glucose metabolism status was determined by the American Diabetes Association criteria based on HbA1c.

Prediabetes status was based in the range of 39-46 mmol/mol and cases of type 2 diabetes were defined as ≥48 mmol/mol [4].

*OR* odds ratio, *CI* confidence interval, *Ref* reference.

### Stratification analysis

Table S1 shows the results from the sub-analysis stratified by sex. We observed similar patterns of BMI trajectory-blood pressure associations in both sexes, with ‘Early high BMI’, ‘Steeper BMI increase’, and ‘Gradual BMI increase’ all associated with elevated blood pressure in both males and females. However, the magnitude of these associations was stronger in males. For instance, in model 3, the ‘Early high BMI’ trajectory was associated with an increase of 8.62 mmHg (95% CI: 6.65, 10.59) in SBP among males, compared to a 7.04 mmHg increase (95% CI: 5.72, 8.36) in females.

The pattern for BMI trajectory-lipid associations differed by sex. For females, the ‘Steeper BMI increase’, ‘Early high BMI’, and ‘Gradual BMI increase’ groups all showed positive associations with total cholesterol and LDL compared to the ‘Stable low BMI’ group in models 2 and 3, consistent with the main analyses. In contrast, for males, the ‘Steeper BMI increase’ group showed negative associations with total cholesterol (β = −0.24 mmol/L, 95% CI: −0.37, −0.11) and LDL (β = −0.18 mmol/L, 95% CI: −0.30, −0.06) in Model 2. After adjusting for medication in Model 3, these negative associations were attenuated and the direction reversed for both total cholesterol (β = 0.17 mmol/L, 95% CI: 0.12, 0.22) and LDL (β = 0.19 mmol/L, 95% CI: 0.14, 0.24).

Among those on lipid or BP-lowering medications, higher BMI trajectories were negatively associated with total cholesterol and LDL compared to the ‘Stable low BMI’ group, the magnitudes of association were stronger in the ‘early high BMI’ trajectory group. There was no association between BMI trajectory groups and blood pressure in the medicated group. For other biomarkers including triglycerides, HDL, HbA1c, and CRP, we observed similar patterns regardless of medication status, with higher BMI trajectory groups showing adverse profiles compared to the ‘Stable low BMI’ reference group. Among participants not taking medications, the results were consistent with our main analyses (Table S2).

### Sensitivity analyses

The magnitudes of association between BMI trajectories and certain biomarkers were attenuated when restricting the analyses to participants older than 50 years. The attenuation was most pronounced for total cholesterol, LDL, SBP, DBP, and slight drop for triglycerides, while the associations with other biomarkers such as HDL, HbA1c, and CRP remained relatively similar between the full sample and the older population (Table S3, Table S4).

## Discussion

By taking advantage of multiple repeated assessments of BMI across a 50-year age period, we identified four distinct BMI trajectories in this large Danish cohort. Groups with consistently high or rapidly increasing BMI were associated with elevated cardiometabolic risk profiles, compared to the ‘Stable low BMI’ weight group. The findings emphasize the importance of considering past weight history for cardiometabolic profiles and suggest that different patterns of BMI elevation evolving during adulthood are associated with varying CVD risk markers.

LCGM was employed to characterize trajectory patterns of repeated measures of BMI over time [8], which can identify heterogeneous developmental patterns while revealing distinct trajectory groups that may influence outcomes [1]. Compared to a single measurement, longitudinal BMI data can incorporate past weight history and reflect dynamic weight changes across the lifespan [29]. A series of studies have identified BMI trajectories in children or adults and associated them with consequent cardiometabolic risk [6, 17, 18, 30–33]. Prior research has examined BMI trajectories within specific life stages across diverse populations, including data from the USA [33], UK [4], Finland [19], Netherlands [29], Canada [34], and China [35]. These investigations delineated BMI trajectories based on age or follow-up duration, yielding varying patterns and group categorizations (ranging from 2 to 6) [19, 36]. Our study identified four trajectories (‘Stable low BMI’; ‘Gradual BMI increase’; ‘Steeper BMI increase’; and ‘Early high BMI’) from early to late adulthood in this Danish population. The variation across studies likely reflects differences in developmental periods captured (growth period, age-range), population characteristics (ethnicity, socioeconomic factors), and methodological approaches to trajectory modeling.

We demonstrated that the heterogeneous BMI trajectories were associated differently with CVD biomarkers. Buscot [19] identified six BMI trajectories from childhood to adulthood in the Cardiovascular Risk in Young Finns Study from 6 to 49 years and found trajectories of worsening or persisting obesity were associated with a 3-fold increased risk of hypertension compared with the ‘stable normal group’, which was similar to our findings. A study from China reported that persistent BMI elevation (BMI ≥ 25 kg/m^2^ progression to ≥30 kg/m^2^) was associated with a detrimental trend in blood pressure in later life (OR = 6.6, 95%CI: 4.5–9.8) [35]. A meta-analysis study also reported the ‘Stable high’ BMI trajectory group displayed a higher risk of hypertension than the ‘sharp-increase’ group (RR = 1.8 versus RR = 1.5) in adults [37]. These results suggest that the cumulative burden of the number of life years spent with higher body weight (≥25 kg/m^2^) is a strong predictor of hypertension risk in late adulthood.

Notably, we found that the rapidly increasing BMI group was more related to lipid profiles than the ‘Early high BMI’ group and ‘Stable low BMI’ group. This rapidly increasing trajectory (‘Steeper BMI increase’) was associated with 6-fold higher odds of dyslipidemia, followed by the ‘Early high BMI’ group and the ‘Gradual BMI increase’ group. The sudden accumulation of body fat may influence metabolic processes, which further increase circulating LDL-cholesterol and triglycerides. Our finding is consistent with the Young Finns study [19], where an excess BMI showed an immediate association with higher levels of LDL and triglycerides. We also found that the ‘steep increase’ group was associated with greater inflammation, as expressed by elevated CRP, than other trajectory groups. Sub-clinical pro-inflammation may be a biological link between BMI gain and cardiometabolic disorders [38–40]. The steeper increase BMI and early high BMI were also strongly associated with elevated levels of HbA1c and a higher risk of prediabetes, consistent with findings from systematic reviews [41].

Early-onset obesity results in significant cumulative exposure to excess adiposity across the lifespan. Rapid weight gain, on the other hand, may overwhelm the body’s adaptive mechanisms, creating a state of metabolic stress. Both early onset and rapid weight gain adversely influence the metabolic profile, including oxidative stress, insulin regulation, and inflammatory response [42]. It is important to note that while adiposity-related changes might explain the observed relationships between BMI trajectories and cardiometabolic biomarkers, these associations might be influenced by other factors such as lifestyle, psychological stress, genetics, medication use, and socioeconomic factors [43, 44]. To sum up, our results suggest that both the age of obesity onset and the rapid transition from normal weight to obesity contribute to the development of cardiovascular risk in late adulthood. These findings should be interpreted within a broader context that acknowledges the complex, multifactorial nature of cardiometabolic health rather than implying direct causality.

Among those on lipid-lowering medications, we observed negative associations between progressing BMI trajectories and lipid profiles (total cholesterol and LDL), with the strongest association in the ‘Early high BMI’ group. This might be explained by medication prescription patterns and adherence as individuals with early high BMI receive earlier and higher dosages of lipid lowering therapy. While medication use modified the associations with lipid profiles, it did not completely mitigate the adverse impact of higher BMI trajectories on other cardiometabolic markers. All weight gain trajectories showed unfavorable profiles for triglycerides, HDL, HbA1c, and CRP regardless of medication status, indicating that pharmacological interventions address specific aspects of cardiometabolic risk but not the broader metabolic dysregulation associated with increasing weight. We observed the pattern for lipids (total cholesterol and LDL) differs by sex. The negative associations between higher BMI trajectories and lipid levels in males, which contrasted with the positive associations seen in females and the main analyses. The results might be explained by mediation use, as 61% of males in our sample were on lipid-lowering drugs compared to only 39% of females. The attenuation of these negative associations in males after medication adjustment suggests that the initially observed negative associations were largely attributable to medication effects. These findings highlight the importance of considering both sex and medication status when examining the relationship between weight trajectories and cardiometabolic outcomes.

While our study demonstrates significant associations between BMI trajectories and cardiometabolic biomarkers, we acknowledge that BMI has inherent limitations as a measure of adiposity. BMI cannot directly measure body fat distribution or distinguish between fat and muscle mass, which are important determinants of metabolic health [45]. Research using alternative adiposity indicators has shown that measures specifically capturing central and visceral adiposity, such as WC, waist-to-height ratio, and body roundness index, may better predict cardiometabolic diseases and mortality outcomes. For example, a Korean cohort study identified five distinct WC trajectory groups among middle-aged adults and found those with high-increasing WC patterns had 5–7 times higher risk for type 2 diabetes compared to the ‘stable-low’ reference group [46]. Similarly, another study demonstrated that central and visceral adiposity indices showed stronger associations with diabetes than BMI among women across different life stages [47]. Future research incorporating multiple adiposity measures may provide more comprehensive insights into the complex relationships between body composition changes and cardiometabolic health.

The main strength of this study is that we capture the complexity of BMI growth trajectories across 50 years, taking into account different numbers of measurements between individuals. Secondly, employing BMI trajectories offers a unique advantage by mitigating potential confounding relationships between BMI and cardiometabolic biomarkers, given its retrospective approach to historical data. We acknowledge the limitations of the study. First of all, there were some missing weight history data thus reducing our sample size as the study criteria was a minimum of 2 BMI records. However, most of the individuals who did not provide the anthropometric information were too young to report any weight history and there were no evident differences in characteristics between those who reported weight history and those who did not (data not shown). Sensitivity analyses including only participants older than 50 years showed similar associations between BMI trajectories and cardiometabolic biomarkers, suggesting our findings are robust. Secondly, our analyses may be affected by residual confounding due to limited information on early-life and life-long potential confounders such as early-life socioeconomic status, alcohol consumption, psychological stress, and other lifestyle factors. These unmeasured variables might not only confound BMI trajectory-CVD risk factor associations but act as effect modifiers, potentially altering the strength of relationships in specific subgroups. Furthermore, medication use represents an important consideration in our analyses. While we adjusted for antihypertensive and lipid-lowering drugs and found they modified associations between BMI trajectories and lipid profiles, we lacked information on other medications (e.g., antidiabetic drugs), medication duration, dosage, or adherence patterns. This limits our ability to establish temporal relationships between trajectory membership and biomarker profiles. Future studies with comprehensive medication histories would help clarify these relationships. Thirdly, weight history was self-reported, which might be subject to recall bias. People tend to underestimate prior weight, particularly those with higher current BMI, which might influence the derived BMI trajectory groups [48]. The magnitude of recall error also tends to increase with the length of the recall period and older age, potentially affecting the accuracy of early adulthood weight reports more than recent ones. However, our validation study in this cohort demonstrated that self-reported BMI is reliable as measured BMI at baseline, which gives some assurance regarding the recalled measures of weight used here [49]. Additionally, while absolute weight values might be subject to recall error, the relative ordering and patterns of weight change over time tend to be more reliably reported, supporting the validity of our trajectory approach even with recalled weights.

## Conclusion

In this large Danish cohort, we found that consistently high or rapidly increasing BMI trajectories were associated with a greater risk of cardiometabolic diseases compared to those at stable low BMI throughout adulthood. Understanding how the BMI trajectories develop across the life course is crucial for gaining insights into their association with health outcomes and offering additional information to clinicians.

## Supplementary information

Life-long Body Mass Index Trajectories and Cardiometabolic Biomarkers-The Danish Diet, Cancer, and Health-Next Generations Cohort

## Data Availability

The data underlying the findings of this study are restricted by the Danish Diet, Cancer and Health Scientific Management Group. Data can be made available from the Danish Cancer Society by following the Data Access Procedures for researchers who meet the criteria for access to sensitive data. The application form can be obtained by contacting DCHdata@cancer.dk.
