# Supplementary material for: Life-long body mass index trajectories and cardiometabolic biomarkers-the Danish diet, cancer, and health-next generations cohort
Source: Int J Obes (Lond). 2025 Aug 22;49(11):2311–9. doi: 10.1038/s41366-025-01882-7 (PMC12583120; doi:10.1038/s41366-025-01882-7)
Supplement: Supplementary file 1 — Life-long Body Mass Index Trajectories and Cardiometabolic Biomarkers-The Danish Diet, Cancer, and Health-Next Generations Cohort [file 41366_2025_1882_MOESM1_ESM.docx]

**Life-long Body Mass Index Trajectories and Cardiometabolic Biomarkers-The Danish Diet, Cancer, and Health-Next Generation Cohort**

Jie Zhang^1,2*^, Christina Andersen^1^, Anja Olsen^1,3^, Jytte Halkjær^3^, Kristina Elin Petersen^3^, Jonas Frey Rosborg Schaarup^1,2^, Christian S Antoniussen^1^, Daniel R Witte^1,2^, Christina C Dahm^1^

1 Department of Public Health, Aarhus University, Aarhus, Denmark;

2 Steno Diabetes Center Aarhus, Aarhus, Denmark;

3 Danish Cancer Institute, Copenhagen, Denmark;

**Table of Contents**

**Figure S1. Participant Workflow in the Analysis of the DCH-NG Cohort**

**Figure S2. Model fit evaluation for cubic models**

**Table S1. Association Between BMI Trajectory Groups and Cardiometabolic Biomarkers in Multivariable Regression Models stratified by sex**

**Table S2. Association Between BMI Trajectory Groups and Cardiometabolic Risk in Multivariable Regression Models for participants stratified by medication**

**Table S3. Association Between BMI Trajectory Groups and Cardiometabolic Biomarkers in Multivariable Regression Models for participants older than 50 years**

**Table S4. Associations Between BMI Trajectory Groups and Cardiometabolic Risk in Logistic Regression Models-older than 50 years**

Descendants identified via CPR

(n=255,608)

Eligible descendants

(n=197,639)

Not possible to invite due to CPR status*

(n=13,875)

Invited by letter

(n=183,764)

Refuse to participate or less than 18 years

(n=138,895)

Agree to participant

(n=44,869)

Did not fulfill questionnaire or have study center assessment

(n=5,315)

Not registered in CRP or not contactable.

Without a valid address in Denmark.

Younger than 18 years old at the time of

recruitment

(n=57,969)

Withdraw consent (n=1)

Pregnant (n=39)

Available with anthropometrics

(n=39,514)

Participants

(n=39,554)

Less than 2 recalled BMI* (n=8,894)

Older than 70 years (n=39)

Included in analyses

(n = 30,581)

Identification

Screening

Included

Figure S1. Participant Workflow in the Analysis of the DCH-NG Cohort


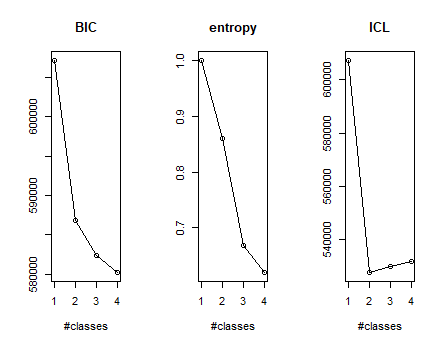


Figure S2. Model fit evaluation for cubic models

Model assessment

BIC: Bayesian Information Criterion; ICL: Integrated Completed Likelihood

**Table S1. Association Between BMI Trajectory Groups and Cardiometabolic Biomarkers in Multivariable Regression Models stratified by sex**

|  | **Models** | **Trajectory groups** | **Triglycerides***  **(mmol/L)** | **Total Cholesterol**  **(mmol/L)** | **HDL**  **(mmol/L)** | **LDL**  **(mmol/L)** | **HbA1c***  **(mmol/mol)** | **CRP***  **(mg/L)** | **Creatinine***  **(μmol/L)** | **SBP**  **(mmHg)** | **DBP**  **(mmHg)** |
| --- | --- | --- | --- | --- | --- | --- | --- | --- | --- | --- | --- |
|  |  |  | **β[95%CI]** | **β[95%CI]** | **β[95%CI]** | **β[95%CI]** | **β[95%CI]** | **β[95%CI]** | **β[95%CI]** | **β[95%CI]** | **β[95%CI]** |
| Female | 1 | Stable Normal | ref | ref | ref | ref | ref | ref | ref | ref | ref |
|  |  | Gradual  BMI increase | **0.09** | **-0.00** | **-0.14** | **0.07** | **0.00** | **0.27** | **0.01** | **1.62** | **1.62** |
|  |  |  | [0.07, 0.11] | [-0.03, 0.03] | [-0.16, -0.12] | [0.04, 0.10] | [-0.00, 0.00] | [0.24, 0.30] | [0.00, 0.02] | [1.07, 2.17] | [1.24, 2.00] |
|  |  | Steeper BMI increase | **0.33** | **0.10** | **-0.34** | **0.24** | **0.03** | **0.88** | **-0.00** | **5.07** | **4.64** |
|  |  |  | [0.31, 0.35] | [0.06, 0.14] | [-0.36, -0.32] | [0.21, 0.27] | [0.03, 0.03] | [0.84, 0.92] | [-0.01, 0.01] | [4.43, 5.71] | [4.20, 5.08] |
|  |  | Early high BMI | **0.26** | **-0.10** | **-0.35** | **0.07** | **0.03** | **0.94** | **-0.03** | **5.04** | **4.82** |
|  |  |  | [0.22, 0.30] | [-0.18, -0.02] | [-0.39, -0.31] | [-0.00, 0.14] | [0.02, 0.04] | [0.86, 1.02] | [-0.04, -0.02] | [3.64, 6.44] | [3.87, 5.77] |
|  | 2 | Stable Normal | ref | ref | ref | ref | ref | ref | ref | ref | ref |
|  |  | Gradual  BMI increase | **0.11** | **0.07** | **-0.13** | **0.13** | **0.01** | **0.29** | **0.01** | **2.66** | **2.17** |
|  |  |  | [0.09, 0.13] | [0.04, 0.10] | [-0.14, -0.12] | [0.10, 0.16] | [0.01, 0.01] | [0.26, 0.32] | [0.00, 0.02] | [2.13, 3.19] | [1.81, 2.53] |
|  |  | Steeper BMI increase | **0.35** | **0.17** | **-0.33** | **0.29** | **0.03** | **0.90** | **-0.00** | **6.16** | **5.22** |
|  |  |  | [0.33, 0.37] | [0.13, 0.21] | [-0.35, -0.31] | [0.26, 0.32] | [0.03, 0.03] | [0.86, 0.94] | [-0.01, 0.01] | [5.55, 6.77] | [4.80, 5.64] |
|  |  | Early high BMI | **0.31** | **0.10** | **-0.31** | **0.21** | **0.04** | **1.00** | **-0.02** | **8.06** | **6.42** |
|  |  |  | [0.27, 0.35] | [0.02, 0.18] | [-0.35, -0.27] | [0.14, 0.28] | [0.03, 0.05] | [0.92, 1.08] | [-0.03, -0.01] | [6.73, 9.39] | [5.50, 7.34] |
|  | 3 | Stable Normal | ref | ref | ref | ref | ref | ref | ref | ref | ref |
|  |  | Gradual  BMI increase | **0.11** | **0.08** | **-0.13** | **0.13** | **0.00** | **0.28** | **0.01** | **2.42** | **2.02** |
|  |  |  | [0.09, 0.13] | [0.05, 0.11] | [-0.14, -0.12] | [0.10, 0.16] | [-0.00, 0.00] | [0.25, 0.31] | [0.00, 0.02] | [1.90, 2.94] | [1.66, 2.38] |
|  |  | Steeper BMI increase | **0.34** | **0.21** | **-0.32** | **0.32** | **0.03** | **0.88** | **-0.00** | **5.36** | **4.76** |
|  |  |  | [0.32, 0.36] | [0.17, 0.25] | [-0.34, -0.30] | [0.29, 0.35] | [0.03, 0.03] | [0.84, 0.92] | [-0.01, 0.01] | [4.75, 5.97] | [4.34, 5.18] |
|  |  | Early high BMI | **0.30** | **0.15** | **-0.30** | **0.25** | **0.04** | **0.96** | **-0.02** | **7.04** | **5.82** |
|  |  |  | [0.26, 0.34] | [0.07, 0.23] | [-0.34, -0.26] | [0.18, 0.32] | [0.03, 0.05] | [0.88, 1.04] | [-0.03, -0.01] | [5.72, 8.36] | [4.90, 6.74] |
| Male | 1 | Stable Normal | ref | ref | ref | ref | ref | ref | ref | ref | ref |
|  |  | Gradual  BMI increase | **0.15** | **0.06** | **-0.14** | **0.08** | **0.01** | **0.16** | **0.02** | **3.01** | **2.23** |
|  |  |  | [0.12, 0.18] | [0.01, 0.11] | [-0.16, -0.12] | [0.04, 0.12] | [0.01, 0.01] | [0.11, 0.21] | [0.01, 0.03] | [2.29, 3.73] | [1.73, 2.73] |
|  |  | Steeper BMI increase | **0.36** | **0.03** | **-0.30** | **0.07** | **0.03** | **0.52** | **0.01** | **6.49** | **5.18** |
|  |  |  | [0.33, 0.39] | [-0.02, 0.08] | [-0.32, -0.28] | [0.02, 0.12] | [0.03, 0.03] | [0.47, 0.57] | [0.00, 0.02] | [5.64, 7.34] | [4.58, 5.78] |
|  |  | Early high BMI | **0.25** | **-0.35** | **-0.32** | **-0.25** | **0.04** | **0.60** | **-0.02** | **5.52** | **4.79** |
|  |  |  | [0.18, 0.32] | [-0.49, -0.21] | [-0.37, -0.27] | [-0.37, -0.13] | [0.03, 0.05] | [0.47, 0.73] | [-0.04, -0.00] | [3.46, 7.58] | [3.35, 6.23] |
|  | 2 | Stable Normal | ref | ref | ref | ref | ref | ref | ref | ref | ref |
|  |  | Gradual  BMI increase | **0.16** | **0.08** | **-0.13** | **0.09** | **0.01** | **0.18** | **0.02** | **3.66** | **2.60** |
|  |  |  | [0.13, 0.19] | [0.03, 0.13] | [-0.15, -0.11] | [0.05, 0.13] | [0.01, 0.01] | [0.13, 0.23] | [0.01, 0.03] | [2.98, 4.34] | [2.11, 3.09] |
|  |  | Steeper BMI increase | **0.29** | **-0.24** | **-0.30** | **-0.18** | **0.06** | **0.71** | **-0.02** | **9.30** | **6.94** |
|  |  |  | [0.22, 0.36] | [-0.37, -0.11] | [-0.35, -0.25] | [-0.30, -0.06] | [0.05, 0.07] | [0.58, 0.84] | [-0.04, -0.00] | [7.33, 11.27] | [5.54, 8.34] |
|  |  | Early high BMI | **0.38** | **0.08** | **-0.29** | **0.10** | **0.04** | **0.58** | **0.01** | **8.28** | **6.20** |
|  |  |  | [0.35, 0.41] | [0.03, 0.13] | [-0.31, -0.27] | [0.05, 0.15] | [0.04, 0.04] | [0.53, 0.63] | [0.00, 0.02] | [7.46, 9.10] | [5.62, 6.78] |
|  | 3 | Stable Normal | ref | ref | ref | ref | ref | ref | ref | ref | ref |
|  |  | Gradual  BMI increase | **0.16** | **0.11** | **-0.13** | **0.12** | **0.01** | **0.18** | **0.02** | **3.52** | **2.53** |
|  |  |  | [0.13, 0.19] | [0.06, 0.16] | [-0.15, -0.11] | [0.08, 0.16] | [0.01, 0.01] | [0.13, 0.23] | [0.01, 0.03] | [2.84, 4.20] | [2.04, 3.02] |
|  |  | Steeper BMI increase | **0.37** | **0.17** | **-0.28** | **0.19** | **0.04** | **0.56** | **0.01** | **7.81** | **5.98** |
|  |  |  | [0.34, 0.40] | [0.12, 0.22] | [-0.30, -0.26] | [0.14, 0.24] | [0.04, 0.04] | [0.51, 0.61] | [0.00, 0.02] | [6.99, 8.63] | [5.40, 6.56] |
|  |  | Early high BMI | **0.27** | **-0.11** | **-0.28** | **-0.05** | **0.05** | **0.69** | **-0.02** | **8.62** | **6.62** |
|  |  |  | [0.20, 0.34] | [-0.24, 0.02] | [-0.33, -0.23] | [-0.17, 0.07] | [0.04, 0.06] | [0.56, 0.82] | [-0.04, -0.00] | [6.65, 10.59] | [5.21, 8.03] |

**Table S2. Association Between BMI Trajectory Groups and Cardiometabolic Biomarkers in Multivariable Regression Models stratified by medication**

| **Stratification by medication** | **Trajectory groups** | **Triglycerides***  **(mmol/L)** | **Total Cholesterol**  **(mmol/L)** | **HDL**  **(mmol/L)** | **LDL**  **(mmol/L)** | **HbA1c***  **(mmol/mol)** | **CRP***  **(mg/L)** | **Creatinine***  **(μmol/L)** | **SBP**  **(mmHg)** | **DBP**  **(mmHg)** |
| --- | --- | --- | --- | --- | --- | --- | --- | --- | --- | --- |
| on Lipid medication | *Stable low BMI* | *ref* |  |  |  |  |  |  |  |  |
|  | Graduate BMI increase | **0.15** | **-0.08** | **-0.14** | **-0.05** | **0.03** | **0.24** | **0.01** | **1.29** | **1.16** |
|  |  | [0.08, 0.22] | [-0.22, 0.05] | [-0.20, -0.09] | [-0.17, 0.06] | [0.01, 0.05] | [0.11, 0.38] | [-0.02, 0.03] | [-0.85, 3.43] | [-0.29, 2.60] |
|  | Steeper BMI increase | **0.36** | **-0.25** | **-0.33** | **-0.17** | **0.10** | **0.66** | **-0.01** | **0.85** | **1.10** |
|  |  | [0.29, 0.44] | [-0.39, -0.11] | [-0.38, -0.27] | [-0.29, -0.05] | [0.08, 0.12] | [0.52, 0.80] | [-0.04, 0.01] | [-1.39, 3.10] | [-0.42, 2.61] |
|  | Early high BMI | **0.33** | **-0.62** | **-0.34** | **-0.49** | **0.14** | **0.91** | **-0.04** | **0.87** | **1.18** |
|  |  | [0.18, 0.48] | [-0.90, -0.34] | [-0.46, -0.22] | [-0.73, -0.24] | [0.11, 0.18] | [0.63, 1.18] | [-0.09, 0.01] | [-3.60, 5.34] | [-1.83, 4.20] |
| not on lipid medication | *Stable low BMI* | ref |  |  |  |  |  |  |  |  |
|  | Graduate BMI increase | **0.12** | **0.09** | **-0.13** | **0.14** | **0.00** | **0.28** | **0.01** | **2.81** | **2.24** |
|  |  | [0.11, 0.14] | [0.06, 0.11] | [-0.14, -0.12] | [0.12, 0.16] | [0.00, 0.01] | [0.25, 0.31] | [0.00, 0.02] | [2.39, 3.22] | [1.95, 2.53] |
|  | Steeper BMI increase | **0.35** | **0.21** | **-0.31** | **0.29** | **0.03** | **0.80** | **0.00** | **7.13** | **5.77** |
|  |  | [0.33, 0.36] | [0.18, 0.24] | [-0.32, -0.30] | [0.26, 0.32] | [0.03, 0.03] | [0.77, 0.83] | [-0.01, 0.01] | [6.63, 7.62] | [5.43, 6.12] |
|  | Early high BMI | **0.29** | **0.07** | **-0.30** | **0.17** | **0.03** | **0.93** | **-0.02** | **8.55** | **6.70** |
|  |  | [0.25, 0.32] | [0.00, 0.14] | [-0.33, -0.27] | [0.11, 0.24] | [0.03, 0.04] | [0.86, 1.00] | [-0.03, -0.01] | [7.42, 9.68] | [5.91, 7.49] |
| on BP-Lowering | Stable Normal | ref |  |  |  |  |  |  |  |  |
|  | Graduate BMI increase | **0.15** | **-0.01** | **-0.16** | **0.06** | **0.01** | **0.37** | **0.01** | **0.60** | **0.80** |
|  |  | [0.10, 0.20] | [-0.11, 0.09] | [-0.20, -0.12] | [-0.03, 0.15] | [0.00, 0.02] | [0.27, 0.47] | [-0.01, 0.03] | [-1.01, 2.22] | [-0.28, 1.87] |
|  | Steeper BMI increase | **0.39** | **-0.11** | **-0.36** | **0.02** | **0.07** | **0.86** | **-0.02** | **1.01** | **1.28** |
|  |  | [0.33, 0.44] | [-0.21, -0.01] | [-0.40, -0.32] | [-0.08, 0.11] | [0.06, 0.08] | [0.76, 0.96] | [-0.04, 0.00] | [-0.65, 2.68] | [0.18, 2.38] |
|  | Early high BMI | **0.28** | **-0.34** | **-0.38** | **-0.18** | **0.10** | **1.06** | **-0.01** | **0.71** | **0.61** |
|  |  | [0.18, 0.37] | [-0.53, -0.14] | [-0.46, -0.30] | [-0.35, 0.01] | [0.08, 0.12] | [0.87, 1.25] | [-0.05, 0.03] | [-2.45, 3.87] | [-1.48, 2.70] |
| not on BP-Lowering | Stable Normal | ref |  |  |  |  |  |  |  |  |
|  | Graduate BMI increase | **0.12** | **0.08** | **-0.13** | **0.13** | **0.00** | **0.26** | **0.01** | **2.71** | **2.18** |
|  |  | [0.11, 0.13] | [0.06, 0.11] | [-0.14, -0.12] | [0.11, 0.16] | [0.01, 0.01] | [0.23, 0.29] | [0.01, 0.02] | [2.30, 3.15] | [1.88, 2.47] |
|  | Steeper BMI increase | **0.34** | **0.20** | **-0.30** | **0.28** | **0.03** | **0.76** | **0.00** | **6.76** | **5.56** |
|  |  | [0.32, 0.36] | [0.17, 0.23] | [-0.31, -0.29] | [0.25, 0.31] | [0.03, 0.03] | [0.73, 0.79] | [-0.00, 0.01] | [6.26, 7.27] | [5.21, 5.92] |
|  | Early high BMI | **0.29** | **0.07** | **-0.29** | **0.16** | **0.03** | **0.87** | **-0.02** | **8.08** | **6.48** |
|  |  | [0.25, 0.33] | [-0.01, 0.14] | [-0.32, -0.25] | [0.09, 0.22] | [0.03, 0.04] | [0.79, 0.95] | [-0.04, -0.01] | [6.91, 9.25] | [5.66, 7.31] |
| on lipid or BP-lowering medicaiton | Stable Normal | ref |  |  |  |  |  |  |  |  |
|  | Graduate BMI increase | **0.14** | **0.00** | **-0.14** | **0.05** | **0.01** | **0.31** | **0.01** | **1.40** | **1.27** |
|  |  | [0.09, 0.18] | [-0.09, 0.09] | [-0.18, -0.11] | [-0.03, 0.13] | [0.00, 0.02] | [0.22, 0.40] | [-0.01, 0.02] | [-0.03, 2.83] | [0.32, 2.23] |
|  | Steeper BMI increase | **0.37** | **-0.09** | **-0.34** | **0.02** | **0.07** | **0.80** | **-0.02** | **1.72** | **1.72** |
|  |  | [0.33, 0.42] | [-0.18, 0.01] | [-0.38, -0.31] | [0.25, 0.31] | [0.06, 0.08] | [0.71, 0.89] | [-0.04, 0.00] | [0.24, 3.21] | [0.73, 2.71] |
|  | Early high BMI | **0.26** | **-0.33** | **-0.34** | **-0.21** | **0.09** | **1.01** | **-0.01** | **1.64** | **1.16** |
|  |  | [0.17, 0.35] | [-0.51, -0.15] | [-0.41, -0.27] | [-0.38, -0.04] | [0.07, 0.11] | [0.83, 1.19] | [-0.04, 0.02] | [-1.26, 4.54] | [-0.78, 3.09] |
| not on lipid or BP-lowering medicaiton | Stable Normal | ref |  |  |  |  |  |  |  |  |
|  | Graduate BMI increase | **0.12** | **0.09** | **-0.13** | **0.14** | **0.00** | **0.27** | **0.01** | **2.66** | **2.15** |
|  |  | [0.11, 0.13] | [0.06, 0.12] | [-0.14, -0.12] | [0.12, 0.16] | [0.01, 0.01] | [0.24, 0.29] | [0.01, 0.02] | [2.24, 3.08] | [1.85, 2.45] |
|  | Steeper BMI increase | **0.34** | **0.23** | **-0.30** | **0.30** | **0.03** | **0.77** | **0.01** | **6.84** | **5.63** |
|  |  | [0.32, 0.35] | [0.19, 0.26] | [-0.31, -0.28] | [0.27, 0.33] | [0.02, 0.03] | [0.74, 0.80] | [0.00, 0.01] | [6.33, 7.35] | [5.27, 5.99] |
|  | Early high BMI | **0.29** | **0.10** | **-0.29** | **0.19** | **0.03** | **0.88** | **-0.02** | **8.18** | **6.57** |
|  |  | [0.25, 0.33] | [0.02, 0.17] | [-0.32, -0.26] | [0.12, 0.26] | [0.02, 0.04] | [0.80, 0.96] | [-0.04, -0.01] | [7.00, 9.37] | [5.74, 7.41] |

*Models adjusted for age, sex, and smoking.

**Table S3. Association Between BMI Trajectory Groups and Cardiometabolic Biomarkers in Multivariable Regression Models-older than 50 years**

| **Models** | **Triglycerides***  **(mmol/L)** | **Total Cholesterol**  **(mmol/L)** | **HDL**  **(mmol/L)** | **LDL**  **(mmol/L)** | **HbA1c***  **(mmol/mol)** | **CRP***  **(mg/L)** | **Creatinine***  **(μmol/L)** | **SBP**  **(mmHg)** | **DBP**  **(mmHg)** | **DBP** |
| --- | --- | --- | --- | --- | --- | --- | --- | --- | --- | --- |
| 1 | Graduate BMI increase | 0.22 | 0.00 | -0.28 | 0.13 | 0.01 | 0.25 | 0.10 | 6.94 | 3.91 |
|  |  | [0.20, 0.24] | [-0.03, 0.03] | [-0.30, -0.26] | [0.10, 0.16] | [0.01, 0.01] | [0.22, 0.28] | [0.09, 0.11] | [6.36, 7.52] | [3.53, 4.29] |
|  | Steeper BMI increase | 0.38 | -0.03 | -0.40 | 0.10 | 0.04 | 0.77 | 0.04 | 8.02 | 5.58 |
|  |  | [0.36, 0.40] | [-0.07, 0.01] | [-0.42, -0.38] | [0.06, 0.14] | [0.04, 0.04] | [0.73, 0.81] | [0.03, 0.05] | [7.28, 8.76] | [5.11, 6.05] |
|  | Early high BMI | 0.28 | -0.19 | -0.34 | -0.06 | 0.04 | 0.87 | -0.02 | 6.23 | 4.77 |
|  |  | [0.22, 0.34] | [-0.31, -0.07] | [-0.39, -0.29] | [-0.17, 0.05] | [0.03, 0.05] | [0.75, 0.99] | [-0.04, 0.00] | [4.16, 8.30] | [3.44, 6.10] |
| 2 | Graduate BMI increase | 0.14 | 0.06 | -0.14 | 0.11 | 0.01 | 0.31 | 0.01 | 2.97 | 2.29 |
|  |  | [0.12, 0.16] | [0.02, 0.10] | [-0.16, -0.12] | [0.08, 0.14] | [0.01, 0.01] | [0.27, 0.35] | [0.00, 0.02] | [2.38, 3.56] | [1.89, 2.69] |
|  | Steeper BMI increase | 0.34 | 0.01 | -0.33 | 0.10 | 0.04 | 0.81 | 0.00 | 6.62 | 4.98 |
|  |  | [0.32, 0.36] | [-0.03, 0.05] | [-0.35, -0.31] | [0.06, 0.14] | [0.04, 0.04] | [0.77, 0.85] | [-0.01, 0.01] | [5.91, 7.33] | [4.51, 5.45] |
|  | Early high BMI | 0.29 | -0.15 | -0.33 | -0.04 | 0.05 | 0.94 | -0.03 | 7.42 | 5.11 |
|  |  | [0.23, 0.35] | [-0.27, -0.03] | [-0.38, -0.28] | [-0.15, 0.07] | [0.04, 0.06] | [0.82, 1.06] | [-0.05, -0.01] | [5.46, 9.38] | [3.80, 6.42] |
| 3 | Graduate BMI increase | 0.14 | 0.09 | -0.14 | 0.14 | 0.01 | 0.30 | 0.01 | 2.69 | 2.14 |
|  |  | [0.12,0.15] | [0.06,0.12] | [-0.16,-0.12] | [0.10,0.17] | [0.00,0.01] | [0.27,0.34] | [0.01,0.02] | [2.10,3.28] | [1.75,2.53] |
|  | Steeper BMI increase | 0.32 | 0.11 | -0.31 | 0.19 | 0.04 | 0.79 | -0.00 | 5.73 | 4.49 |
|  |  | [0.30,0.35] | [0.07,0.15] | [-0.33,-0.29] | [0.16,0.23] | [0.03,0.04] | [0.75,0.83] | [-0.01,0.00] | [5.01,6.44] | [4.01,4.97] |
|  | Early high BMI | 0.26 | -0.02 | -0.31 | 0.08 | 0.04 | 0.90 | -0.03 | 6.16 | 4.42 |
|  |  | [0.20,0.32] | [-0.13,0.09] | [-0.36,-0.26] | [-0.02,0.19] | [0.03,0.05] | [0.79,1.02] | [-0.05,-0.01] | [4.21,8.11] | [3.12,5.73] |

Multivariable linear models between BMI trajectory groups and cardiometabolic biomarkers, with stable normal weight as reference group

Abbreviations: BMI, body mass index; CI, confidence interval, CRP**,** C-reactive Protein; DBP, diastolic blood pressure; HbA1c, hemoglobin A1c; HDL, high-density HDL; LDL, low-density lipoprotein; SBP, systolic blood pressure; OW, overweight; OB, obesity

Mode1 crude model; Model 2 was adjusted for age, sex, and smoking; Model 3 was adjusted for age, sex, and smoking and medication; *log-transformed

**Table S4. Associations Between BMI Trajectory Groups and Cardiometabolic Risk in Logistic Regression Models-older than 50 years**

|  |  |  |  |  |  |  |  |  |  |
| --- | --- | --- | --- | --- | --- | --- | --- | --- | --- |
|  | **Model 1** | | | **Model 2** | | | **Model 3** | | |
| **Hypertension** | OR | 95% CI | | OR | 95% CI | | OR | 95% CI | |
| Stable low BMI | Ref. |  |  | Ref. |  |  | Ref. |  |  |
| Graduate BMI increase | 1.77 | 1.64 | 1.90 | 1.38 | 1.28 | 1.50 | 1.35 | 1.24 | 1.46 |
| Steeper BMI increase | 2.43 | 2.22 | 2.67 | 2.29 | 2.08 | 2.51 | 2.10 | 1.91 | 2.31 |
| Early high BMI | 2.13 | 1.67 | 2.72 | 2.37 | 1.85 | 3.04 | 2.09 | 1.63 | 2.70 |
| **Dyslipidemia** | |  |  |  |  |  |  |  |  |
| Stable low BMI | Ref. |  |  | Ref. | |  | Ref. | |  |
| Graduate BMI increase | 2.25 | 1.89 | 2.67 | 2.19 | 1.84 | 2.62 | 2.21 | 1.85 | 2.64 |
| Steeper BMI increase | 5.18 | 4.35 | 6.17 | 5.14 | 4.30 | 6.14 | 5.34 | 4.46 | 6.40 |
| Early high BMI | 3.42 | 2.25 | 5.19 | 3.54 | 2.32 | 5.39 | 3.69 | 2.41 | 5.63 |
| **Pre-diabetes and diabetes** | | |  |  |  |  |  |  |  |
| Stable low BMI | Ref. |  |  | Ref. |  |  | Ref. |  |  |
| Graduate BMI increase | 2.22 | 1.71 | 2.88 | 1.85 | 1.41 | 2.43 | 1.67 | 1.27 | 2.19 |
| Steeper BMI increase | 7.83 | 6.08 | 10.08 | 7.95 | 6.15 | 10.29 | 5.98 | 4.59 | 7.81 |
| Early high BMI | 9.75 | 6.33 | 15.00 | 12.58 | 8.11 | 19.52 | 8.62 | 5.43 | 13.69 |

Abbreviations: Normal-OW=Normal to Overweight; OW-OB=Overweight to obesity: Normal-OW-OB=Nonlinear normal-overweight-obesity; OR, odds ratio; CI, confidence interval; Ref, reference

Model 1 was crude model; Model 2 was adjusted for age, sex, and smoking; Model 3 was adjusted for age, sex, and smoking and medication

Hypertension was defined as systolic/diastolic BP ≥140/90 mmHg, self-reported doctor diagnosis of hypertension, or use of BP-lowering medication. Dyslipidemia was defined as having triglycerides >2.0 mmol/l or HDL <1.0 mmol/l based upon the recommendations by the National Heart Foundation(2) and the Australian Diabetes Society(3). Glucose metabolism status was determined by the American Diabetes Association criteria based on HbA1c.

Prediabetes status was based in the range of 39-46 mmol/mol and cases of type 2 diabetes were defined as ≥48 mmol/mol(4).

Reference

1. Panel E. Executive summary of the clinical guidelines on the identification, evaluation, and treatment of overweight and obesity in adults. Arch Intern Med. 1998;158:1855-67.

2. Atherton JJ, Sindone A, De Pasquale CG, Driscoll A, MacDonald PS, Hopper I, et al. National Heart Foundation of Australia and Cardiac Society of Australia and New Zealand: guidelines for the prevention, detection, and management of heart failure in Australia 2018. Heart, Lung and Circulation. 2018;27(10):1123-208.

3. Best JD, Jerums G, Newnham HH, O'BRIEN RC. Diabetic dyslipidaemia: Australian Diabetes Society position statement. Medical journal of Australia. 1995;162(2):91-3.

4. Gillett MJ. International expert committee report on the role of the A1c assay in the diagnosis of diabetes: diabetes care 2009; 32 (7): 1327–1334. The Clinical Biochemist Reviews. 2009;30(4):197.
